# Characteristics and first treatment dose of Dutch patients (12-60 years old) receiving prescriptions for asthma and initiating inhaled corticosteroids (ICS) therapy as either extra-fine (EF) ciclesonide or standard-particle (SP)-ICS

**DOI:** 10.1186/1939-4551-8-S1-A259

**Published:** 2015-04-08

**Authors:** Daniela Van Eickles, David Price, Javaria Mona Khalid, Ron Herings, Jetty Overbeek, Julie Von Ziegenweidt, Muzammil Ali, Cristiana Miglio

**Affiliations:** 1 Takeda International, UK; 2 Academic Primary Care, University of Aberdeen, UK; 3 Research in Real Life, UK; 4 Pharmo Institute for Drug Outcomes Research, the Netherlands

## Background

Asthma management guidelines suggest little difference between EF and SP-ICS other than potency and therefore EF-ICS should be used at same dose as fluticasone (FP) and half the dose of SP-beclomethasone (BDP). Cohort studies suggest EF-BDP patients can achieve better asthma control than FP patients at lower doses. We compared baseline characteristics and first prescribed doses of patients initiating ciclesonide vs. SP-ICS.

## Methods

Data from the PHARMO Database Network (pharmacy and hospital discharge records) on patients (12-60 years old) with ≥2 prescriptions for asthma therapy (2005-2012) were compared over 1 year before initiating ciclesonide vs SP-ICS. Co-morbidities were evaluated over 1 year before and after ICS initiation. To avoid inclusion of potential COPD patients, those >60 years old and those using long-acting muscarinic antagonists were excluded. Sex and age at ICS initiation; initial ICS doses (actual prescribed doses); short-acting β2-agonists (SABA) use (year before initiation); prescriptions for acute oral steroids and overall asthma control (no hospital admissions, no acute oral steroids and ≤200mcg/day salbutamol) in the year prior and including initiation date; and prescriptions of drugs for treating co-morbidities (year before and after initiation) were compared using t-test/chi-square test (p<0.05).

## Results

Of 4,064 patients, 34% initiated therapy as ciclesonide and 66% as SP-ICS, with same proportion of males (36%). Differences (p<0.001, unless otherwise specified) for ciclesonide vs. SP-ICS were: mean(±SD) age (43±13 vs. 38±14 years); median(Inter Quartile Range) initial ICS doses 160(160-160) vs. 500(250-500) μg; proportion of patients not on SABA (72% vs. 57%) and on SABA daily dosage between 1-100 μg/day (21% vs. 29%), 101-200 μg/day (5% vs. 9%) and >200 μg/day (2% vs. 6%); proportion of patients not prescribed acute oral steroids (90% vs. 88%, p=0.016) and with controlled asthma (87% vs. 82%); proportion of patients prescribed nasal (44% vs. 38%) and topical (31% vs. 28%) steroid preparations, proton-pump inhibitors (41% vs. 29%) and cardiac diseases or hypertension drugs (28% vs. 21%).

## Conclusions

For comparable asthma control and similar prevalence of co-morbidities, patients were prescribed triple the dose of SP-ICS versus ciclesonide. Further to this analysis, the effects on asthma control in the year following ICS initiation will be investigated.

